# Exploring Nurses' Experiences in Caring for Medical‐Psychiatric Comorbid Patients: A Qualitative Interview Study

**DOI:** 10.1111/inm.70156

**Published:** 2025-10-21

**Authors:** Ruth Jong, Sharon van den Berg, Sigrid C. J. M. Vervoort, Monique van Dijk

**Affiliations:** ^1^ Department of Internal Medicine, Nursing Science Section Erasmus University Medical Center Rotterdam the Netherlands; ^2^ Department of Psychiatry Erasmus MC, University Medical Center Rotterdam Rotterdam the Netherlands; ^3^ Department of General Practice and Nursing Science, Julius Center for Health Sciences and Primary Care and Clinical Health Sciences University Medical Center Utrecht and Utrecht University Utrecht the Netherlands

**Keywords:** general hospitals, integrated delivery of healthcare, medical‐psychiatric comorbidities, mental health, nurse experiences

## Abstract

Approximately one‐third of patients who visit general hospitals have a mental disorder, which is associated with poorer outcomes and higher costs. Caring for these patients can be challenging for nurses, particularly when behaviours such as aggression, wandering, or lack of cooperation occur. To explore how nurses experience caring for patients with medical‐psychiatric comorbidities in general hospital wards a qualitative study using semi‐structured interviews was conducted. Sixteen registered nurses with varying experience from eight wards in a Dutch university medical centre participated. Data were analysed inductively using Braun and Clarke's thematic analysis. Nurses' experiences were captured in three themes: *managing emotions*, *adapting to contextual factors* and *building competence*. *Managing emotions* reflected feelings ranging from frustration to compassion, and strategies such as peer support and rationalisation to cope. *Adapting to contextual factors* highlighted challenges including time pressures, competing demands and environments ill‐suited to mental health needs. *Building competence* involved learning through experience and emphasised the need for education and multidisciplinary collaboration. Nurses' challenges in caring for patients with medical‐psychiatric comorbidities stem less from the patient's behaviour itself than from limited training, organisational structures and systemic barriers. Caring for these patients requires more than individual coping strategies; organisational, educational and interprofessional support are essential to improve care delivery and nurse well‐being. Simulation‐based training, structured reflection and interprofessional collaboration may strengthen competence and confidence, while organisational adjustments could foster safer and more person‐centred care.

## 1 Introduction

Mental disorders are highly prevalent worldwide, affecting one in eight people (World Health Organization 2022). The World Health Organization (2022) defines a mental disorder as “a clinically significant disturbance in an individual's cognition, emotional regulation, or behaviour”. Within general hospital wards, psychiatric comorbidities are particularly common: approximately one‐third of admitted patients have a diagnosable mental disorder (Mather et al. 2014; van Niekerk et al. 2022). These comorbidities are consistently associated with worse outcomes, including extended lengths of stay, higher readmission rates, greater morbidity and higher healthcare costs compared to patients without psychiatric comorbidity (Jansen et al. 2018).

Many patients with medical illnesses and psychiatric comorbidity are underdiagnosed and undertreated, which may partly explain the increased morbidity and mortality rates in this population (Mather et al. 2014). Evidence from two integrative reviews indicates that stigma, negative attitudes and limited mental health literacy among healthcare professionals contribute to poor outcomes, while stigma and anticipated discrimination may delay patients' help‐seeking (Alexander et al. 2016; de Jacq et al. 2016; Giandinoto and Edward 2015).

Optimal care and support of patients with psychiatric comorbidity in hospital settings is therefore crucial.

In clinical practice, patients with psychiatric comorbidities may exhibit behaviours such as verbal or physical aggression, lack of cooperation or wandering (Chou and Tseng 2020; Derblom et al. 2022; Giandinoto and Edward 2015; Holmberg et al. 2020; Peart et al. 2023). These behaviours can be understood as expressions of emotional distress or attempts to communicate unmet needs. While often perceived by staff as difficult to cope with, such behaviours frequently demand a disproportionate allocation of resources, including time, personnel and medication (Derblom et al. 2022; Holmberg et al. 2020; Peart et al. 2023; Zetterberg et al. 2022). Providing care in these situations places additional demands on nurses, especially when adequate training, support or organisational resources are lacking.

## 2 Background

Previous research has highlighted several challenges faced by nurses in caring for patients with psychiatric comorbidities. Nurses often experience frustration, fear and emotional exhaustion when caring for patients with psychiatric comorbidities (Beks et al. 2018; Chou and Tseng 2020; Marynowski‐Traczyk and Broadbent 2011; Plant and White 2013). Many of them have received little or no formal mental healthcare training and therefore may fail to recognise and adequately respond to psychiatric symptoms (Giandinoto and Edward 2015; Poggenpoel et al. 2011). Studies conducted in acute care settings indicate that nurses often express concerns about the safety, privacy and adequacy of facilities in hospital settings (Aebi et al. 2021; Chou and Tseng 2020; Giandinoto and Edward 2015). In general hospital wards, open ward structures may further complicate care by contributing to patient disorientation and wandering behaviours, while limited staffing ratios further constrain the capacity to respond to patients' complex needs.

Previous research has shown that nurses in general hospital wards often struggle with caring for patients with psychiatric comorbidities. Foye et al. interviewed general ward staff from a UK hospital, who reported that they often distanced themselves from mental healthcare due to limited training, fear, emotional strain and a lack of leadership. Similarly, a qualitative study by Poggenpoel et al. (2011) conducted in South Africa found that nurses on medical wards attributed their feelings of frustration, unhappiness and fear to insufficient knowledge and skills. These findings underline that the challenges are not limited to a specific healthcare system but are reported across diverse and international contexts. In large urban centres in the Netherlands, healthcare workers must deal with patients with psychiatric comorbidities. A report by the Netherlands Court of Audit estimated that thousands of people with severe psychiatric disorders wait 4 months or longer for treatment (Netherlands Court of Audit 2020), resulting in increased admissions of patients with complex psychiatric conditions to general hospitals. Consequently, general hospital nurses increasingly encounter patients with complex psychiatric needs, often without sufficient preparation or systemic support. Dutch nursing curricula also provide limited education on psychiatric care (Huisman‐De Waal et al. 2019). Moreover, urban hospitals frequently care for culturally diverse patients, for whom migration‐related stress, socio‐economic deprivation and language barriers may compound mental health challenges (Ozgen et al. 2022). These systemic and contextual factors converge to create a particularly complex environment for general ward nurses.

No previous studies have examined how nurses in Dutch general hospital wards experience caring for patients with psychiatric comorbidities. Understanding these experiences is essential for enhancing nurse competency, reducing burnout, fostering a supportive work environment and ultimately improving patient outcomes. The research question guiding this study is: How do nurses in general hospital wards experience caring for patients with medical‐psychiatric comorbidities?

## 3 Method

### 3.1 Design

An experiential qualitative design was employed. An experiential design, rooted in a phenomenological orientation, focuses on exploring participants' experiences, views and perspectives, practices and behaviours, rather than testing theory or causal explanations (Braun and Clarke 2013, 2022).

Participants were included until inductive thematic saturation was reached, meaning that new data no longer produced substantially new codes or themes during the analysis (Saunders et al. 2018).

### 3.2 Setting and Participants

The study was conducted at a Dutch university medical centre, known for its complex patient population and diverse medical specialties. The focus of the study was on general wards where nurses provide care for patients with various medical conditions, including medical‐psychiatric comorbidities. Eligible participants were nurses from internal medical, surgical or combined wards experienced in managing patients with medical‐psychiatric comorbidities. Nurses from emergency departments, intensive care units, psychiatric wards, paediatric wards and student nurses were excluded due to differences in patient population, nurse‐to‐patient ratios and responsibilities.

Purposive sampling aimed to achieve maximum variation in sex, age, years of experience and ward type, as these factors may influence nursing perspectives (Fernandez‐Feito et al. 2019). Managers of eligible wards were emailed information about the study's aim, procedures and ethical considerations and shared this information with their nursing staff. Interested nurses contacted the researcher directly.

Sixteen nurses from eight wards agreed to participate, and no participants withdrew during the study. The sample included three males and 13 females, all registered nurses without postgraduate psychiatric qualifications. Nursing experience ranged from 2 to 34 years (see Table 1 for detailed participant characteristics).

**TABLE 1:** Participants characteristics.

	*N* = 16
Sex	
Male	3 (18.75%)
Female	13 (81.25%)
Age groups (years)	
20–29	8 (50%)
30–39	2 (12.5%)
40–49	3 (18.75%)
> 50	3 (18.75%)
Experience as a nurse (years)	
1–5	6 (37.5%)
6–10	3 (18.75%)
11–15	2 (12.5%)
16–20	2 (12.5%)
> 20	3 (18.75%)
Ward	
Internal medicine ward	6 (37.5%)
Surgical ward	3 (18.75%)
Combined ward	7 (43.75%)

### 3.3 Data Collection

Semi‐structured interviews were conducted using an interview guide, which was developed based on the literature (Aebi et al. 2021; Chou and Tseng 2020) and expert opinion of the author M.v.D, a female professor in nursing science. It included questions about socio‐demographic information, participants' experiences, perceived barriers and facilitators and suggestions for improving care (Table A1). All interviews began with the opening question: “What experiences do you have in caring for patients with a medical‐psychiatric comorbidity?” All interviews were conducted by RJ, a female master's student in nursing science who received training in qualitative research methods and interview techniques. She also conducted a pilot interview to obtain feedback from M.v.D on interview techniques, but the pilot interview was not included in the final analysis. Interviews were intended to be conducted face‐to‐face in a quiet place at the hospital. All interviews took place face‐to‐face between February and April 2024, except for one conducted via FaceTime at the participant's request. Only the participants and the interviewer were present during the interviews. The interviews had a mean duration of 27 min and a range of 17–41 min. No repeat interviews were conducted. Before each interview, the interviewer introduced herself, explained the study rationale and emphasised that no judgements would be made. All interviews were audio recorded. Field notes were taken afterwards, including memos on key themes, interviewer reflections and emerging patterns (Braun and Clarke 2013). Data collection and analysis were conducted iteratively, with intermediate analysis leading to adjustments in the interview guide.

### 3.4 Data Analysis

Descriptive statistics were used to summarise participant characteristics and were performed with IBM SPSS Statistics for Windows, Version 27.0 (Armonk, NY: IBM Corp). All interviews were transcribed verbatim using Microsoft Word and analysed using NVivo version 12.0.

Braun and Clarke's thematic analysis was employed to examine the qualitative data, capturing participants' perspectives and identifying patterns (Braun and Clarke 2022). An inductive approach ensured the findings were data‐driven rather than theory‐based (Braun and Clarke 2022).

The thematic analysis involved six iterative phases, which continued until no new codes or themes emerged. In the first phase, both researchers (R.J. and M.v.D.) familiarised themselves with the data by repeatedly reading the transcripts, searching for meanings and patterns and noting aspects of interest.

In the second phase, R.J. conducted an initial cycle of coding to develop a coding framework (Table A2). Codes were defined as “an analytic tool and output that captures an analytic insight from the researcher's systematic engagement with their data” (Braun and Clarke 2022).

In the third phase, codes were grouped into potential themes and subthemes based on conceptual similarity. A theme was defined as “a shared, multi‐faceted meaning, patterned across the dataset, that includes related insights unified by a central idea” (Braun and Clarke 2022). Different codes were sorted into potential themes and subthemes reflecting experiences of caring for patients with medical‐psychiatric comorbidity. The initial coding framework (Table A2) captured the full range of ideas expressed by participants. This coding framework served as the basis for developing themes. Some codes correspond directly to final subthemes (e.g., ‘dividing time and attention’ and ‘time pressure and inequity in attention’), while others were merged or reframed (e.g., ‘personality’ contributed to ‘managing emotions’). A few codes, such as ‘family and relatives’, appeared infrequently and across varied contexts. Relevant elements were integrated into broader themes, but they did not emerge strongly enough to form a separate subtheme.

In the fourth phase, themes were refined and discussed during a joint meeting of the researchers and peer‐reviewed by a third researcher (S.V.), a nurse scientist experienced with conducting qualitative research. A thematic map was created to visually represent the themes and their relationships (Braun and Clarke 2022).

In the fifth phase, themes were defined and further refined by revisiting the data extracts for each theme. Each theme was organised into a cohesive and logically structured presentation accompanied by a corresponding narrative. Final theme names were selected in collaboration with the research team, and each theme was briefly summarised and explained. Through discussions between researchers R.J. and M.v.D. and a re‐reading of the transcripts, the themes were confirmed. A member check of the synthesised analysed data was performed, ensuring that descriptions and interpretations accurately reflected participant experiences and could be recognised by others with similar experiences. Of the 16 participants invited for the member check, one participant responded, indicating that the described results accurately reflected their experiences.

In the sixth and last phase, the analysis was written up comprehensively. Representative quotes from participants are included to illustrate themes and subthemes.

### 3.5 Rigour

Reporting of this study followed the Consolidated Criteria for Reporting Qualitative Research (COREQ) (Tong et al. 2007). The study used Lincoln and Guba's model of trustworthiness to ensure rigour. This model encompasses four elements: dependability, credibility, confirmability and transferability (Guba and Lincoln 1989). Prior to the study, a pilot interview was conducted to test and refine the interview guide and to enhance dependability and credibility. Throughout the study, a reflexive journal supported by field notes and observations was maintained to uphold dependability and confirmability (Nowell et al. 2017). Peer review was employed through regular meetings to discuss and monitor the study's progress, thereby enhancing dependability (Nowell et al. 2017). To strengthen credibility, participants were invited to review the synthesised data as a member check (Birt et al. 2016; Nowell et al. 2017; Sandelowski 2000). One participant provided feedback which was treated as an additional perspective rather than a validation of all findings, given the limited response. Transferability was supported by providing a description of the study context and participant characteristics.

### 3.6 Ethics

The study was conducted in accordance with the Declaration of Helsinki (World Medical Association 2013), and the European Union's General Data Protection Regulation (GDPR). The hospital's Medical Ethics Review Committee determined that the study did not fall within the scope of the Medical Research Involving Human Subjects Act (MEC‐2024‐0010). All participants received detailed written and verbal information about the study aim, procedures, confidentiality and their right to withdraw at any time without consequences. Participants provided written informed consent before participation. Participants were informed that the study findings could be published in a scientific journal. To protect privacy, participants' names were coded and data were de‐identified.

## 4 Findings

Nurses' experiences of caring for patients with medical‐psychiatric comorbidities were categorised into three main themes: (1) Managing emotions, (2) Adapting to contextual factors and (3) Building competence. Seven subthemes further detail these main themes (Table 2).

**TABLE 2:** Overview of themes and sub‐themes.

Theme	Subtheme
Managing emotions	Struggling with various emotions Rationalising and coping with emotions
Adapting to contextual factors	Time pressure and inequity in attention Physical environment influences quality of care
Building competence	Closing knowledge gaps Empowering through experience Prioritising multidisciplinary collaboration

### 4.1 Theme 1: Managing Emotions

Nurses experienced a wide range of emotions, such as frustration, uncertainty and compassion, when caring for patients with medical‐psychiatric comorbidities. To cope, nurses rationalised patients' behaviour, sought peer support and emphasised teamwork as essential.

#### 4.1.1 Struggling With Various Emotions

In general, nurses experienced various emotions when caring for patients with medical‐psychiatric comorbidities. They reported frustration and, at times, irritation, particularly when faced with challenging behaviours or repetitive questions from patients. Despite their sincere intent to deliver quality care, nurses often felt uncertain about the optimal approach. Some worried that their actions might trigger challenging behaviour in patients.

Then I think, ‘what is this? Is the patient really like this, or… why me? Is it me? Am I the one who is triggering this behaviour, or something? Or what is happening here? (Participant 4)

Nurses emphasised that they tended to prioritise physiological symptoms over mental health problems, because patients are primarily admitted for physical conditions. As a result, mental health received limited attention, which often contributed to feelings of frustration and disappointment among nurses, as they felt inadequate in addressing patients' needs. Over time, this contributed to a sense of powerlessness, as well as feelings of inadequacy and self‐doubt.

Yes, when the medical aspect is prominent, then I feel capable of that, but as soon as I need to delve deeply into the mental aspect, I do not feel capable of that. (Participant 2)

Nurses also expressed experiencing uncertainty when deciding on restrictive interventions, such as physical restraints or tent beds. While recognising these measures as sometimes necessary for safety, they remained concerned about potential physical and psychological harm. Nurses described such interventions as sometimes ‘inhumane’ and ‘degrading’, reflecting a strong sense of empathy and inner conflict in managing these dilemmas.

That's not acceptable, of course. So, what happens then? You restrain someone, but that makes her mental state worse, and well, also aggressive… (Participant 5)

#### 4.1.2 Rationalising and Coping with Motions

Nurses indicated that they empathised more with patients whose mental health issues stemmed from physical illness or medication, compared to those with primary psychiatric disorders. They described a tendency to distinguish between these groups based on the perceived cause, with physical conditions evoking more compassion. Reframing patients' challenging behaviour as illness‐related helped nurses cope emotionally.

Sometimes I find myself feeling a bit agitated, I'm kind of irritated too. (…) Then I try to rationalize it like, ‘okay, well, it's probably because of something underlying that you're reacting like this'. (Participant 7)

In contrast, behaviours perceived as intentional, such as demanding or manipulative attitudes, were harder for nurses to cope with. Such behaviours were commonly associated with psychiatric disorders, rather than with patients whose psychiatric symptoms were perceived as secondary to a physical illness. When nurses felt their efforts were unappreciated or never enough, it often led to frustration and emotional strain.

That's frustrating. You feel like you're doing your best for a patient, but it's never enough. And it's not fun when it doesn't work out. (Participant 1)

Consulting colleagues was a commonly used strategy to cope with feelings of agitation, irritation or insecurity. Nurses emphasised the value of team working in navigating challenging situations. They frequently relied on conversations with colleagues to process emotional experiences. All nurses appreciated having time and space within the team to discuss both emotional and practical challenges. Alternating tasks with colleagues was also seen as an important way to reduce stress, benefitting both nurses and patients.

Together you are strong. Of course, it shouldn't become a situation where the patient feels it's two against one, but I do appreciate being able to discuss it with a colleague. And I find it comforting to handle it together. With two people, you feel more at ease. (Participant 11)

### 4.2 Theme 2: Adapting to Contextual Factors

Nurses described how structural and situational conditions influenced their ability to care for patients with medical‐psychiatric comorbidities. These contextual challenges included time pressures, the need to balance attention between all patients and environmental features of the ward that sometimes limited safe and effective care.

#### 4.2.1 Time Pressure and Inequity in Attention

Nurses reported difficulties in allocating sufficient time and attention to care for patients with medical‐psychiatric comorbidities while also ensuring timely care for other patients. Attending to patients' psychological and behavioural needs often required extended time, which they perceived as detracting from care for other patients.

And often, when you're just busy with a patient, yeah, de‐escalating, then it is at the expense of all those other patients you have to give medication to, nebulize, mobilize… It's at their expense. (Participant 6)

Concurrently, nurses recognised that neglecting the mental aspect of medical‐psychiatric patients may result in inadequate provision of care, sometimes leading to asking for a transfer of patients to the medical‐psychiatric unit. Additionally, nurses were concerned about the potentially harmful or offensive behaviour of medical‐psychiatric comorbid patients towards other patients, such as wandering into rooms, using inappropriate language or making noise and felt responsible for protecting other patients.

Sometimes the patient walks down the hallway undressed, naked, and then you think, ‘Oh, no…’, […] Other patients see that too, and visiting hours last until eight o'clock. So, you're running around, trying to keep everything in order, but it's actually hard to oversee in such moments. (Participant 2)

#### 4.2.2 Physical Environment Influences Quality of Care

The open ward layout was described as unsuitable for patients prone to wandering, often leading to disorientation. Nurses emphasised that private rooms could reduce overstimulation and improve safety, but that the former shared rooms allowed for better patient supervision. Removing potentially harmful objects from rooms for patients with a history of violence or suicidal behaviour was seen as a necessary step for improving both patient safety and nurses' peace of mind.

We once had a patient who started wandering and entering other people's rooms, even lying on their beds. He was very restless, and it was impossible to leave him alone. (Participant 16)

### 4.3 Theme 3: Building Competence

Nurses sought ways to improve the psychiatric component of care. While many felt underprepared due to limited training, they emphasised that learning through practice and drawing on multidisciplinary expertise helped them to respond more effectively to challenging behaviour.

#### 4.3.1 Closing Knowledge Gaps

Most nurses acknowledged having limited knowledge and experience in caring for patients with medical‐psychiatric comorbidity. They partly attributed this to the workplace culture and training programmes that focused on the physical aspects of nursing. They also reported a lack of information and practical resources on effective strategies for addressing psychiatric symptoms and challenging behaviours.

…but there is little known about what resources we have regarding these kinds of people (…) we rather try to resolve that within the team itself, than being fully aware, I think, of the resources we can use. (Participant 3)

Most nurses expressed a preference for standardised plans or protocols specifically addressing psychiatric comorbidities. Several nurses indicated that specialised training in mental healthcare tailored to general ward settings would improve their confidence and skills. Some nurses found interactive or simulation‐based training more engaging and relevant to daily practice than clinical lessons.

I think something interactive. I think we should make it fun, because the clinical lesson is often so overused, so by the end of your shift, nobody feels like it anymore. No, so something interactive, like a simulation‐type lesson, or something with a training actor. (Participant 8)

#### 4.3.2 Empowering Through Experience

According to nurses, experience plays a significant role in caring for a patient with medical‐psychiatric comorbidities. Nurses noted that their level of experience correlated with their ability to contextualise and address challenging behaviour. Through their experience with patients exhibiting challenging behaviour, nurses gradually learned to communicate their boundaries more effectively. Moreover, they became increasingly able to gain a professional distance from the patients' behaviours.

Over time… the more patients like that you have, the more you learn how to handle it, and the better it goes. (Participant 12)

Nurses also indicated that their experience aided them in advocating for the interests of their patients and fellow nurses. They mentioned that their experience allowed them to confidently address concerns when a physician showed little inclination to address disruptive behaviour. Simultaneously, experienced nurses wondered whether less experienced nurses might not yet feel confident enough to address their concerns to the physician.

Sometimes it [the collaboration with the physician] is so frustrating, and I think ‘seriously?’ and I do mention it to the physician, like ‘funny thing to say, but come stand here and deal with it all the time.’ (…) And I wonder about the novice nurses, to what extent do they do that? They'll just say ‘yes, doctor, yes'. (Participant 4)

#### 4.3.3 Prioritising Multidisciplinary Collaboration

Nurses highlighted the crucial role of collaboration with physicians and psychiatric specialists in providing effective care for patients with medical‐psychiatric comorbidity. Nurses observed that physicians' expertise varied, just as their willingness to seek psychiatric consultations. For example, nurses noted that collaboration with physicians was notably smoother in cases of delirium, where physicians were more familiar with protocols and treatment options.

In other situations, nurses felt the need to actively initiate discussions with physicians about patient behaviour or mental health concerns, including suggesting psychiatric consultation or medication adjustments. While some nurses felt heard and supported by physicians, others experienced a lack of understanding regarding how patient behaviour impacts nursing care.

They see the patient for one minute, we have the patient 24/7, and I don't always get the feeling that they know what it's like for us to deal with it all day long. (Participant 12)

The involvement of psychiatric consultation teams, consisting of a psychiatrist and a nurse practitioner was highly valued. Nurses appreciated their patient‐centred advice and found their reports useful in gaining deeper insights into patients' mental health status.

And I also find it quite nice to read when they visited, to read their reference. (…) They pay attention to very different things, and when I read that, I think, ‘Oh, right, yes.’ (…) It's clarifying to read that. (Participant 9)

Teamwork within nursing staff was emphasised as crucial in managing patients with medical‐psychiatric comorbidities. Most nurses attempted to handle situations within the team, indicating a willingness and preparedness to address challenges. Some nurses also expressed a desire to collaborate more closely with nurses from the medical‐psychiatric unit, valuing their expertise and practical advice.

So, I think we should all, if you think, ‘I have some questions’, just contact the nursing staff from the MPU [medical psychiatric unit] and ask for their expertise. They deal with these things much more often. (Participant 7)

## 5 Discussion

This study offers insight into nurses' experiences caring for patients with medical‐psychiatric comorbidities in general hospital wards. Three main themes were identified: managing emotions, adapting to contextual factors and building competence. Together, these findings suggest that the challenges nurses encounter are not only related to patient behaviour but also to organisational and educational structures that shape how care is delivered.

Participants described a wide range of emotions when caring for patients with medical‐psychiatric comorbidities, from frustration and insecurity to compassion and empathy. Previous research has also documented such feelings among nurses in both emergency and general hospital settings (Beks et al. 2018; Derblom et al. 2022; Holmberg et al. 2020; Peart et al. 2023; Poggenpoel et al. 2011). Participants in this study generally felt more confident in addressing physical over mental healthcare, a finding that mirrors results from acute and medical care wards (Derblom et al. 2022; Peart et al. 2023; Poggenpoel et al. 2011). This pattern reflects the biomedical focus of hospital culture and contributes to role conflict, leaving participants frustrated by their inability to adequately address mental health needs. Emotional strain was further heightened by the responsibility they felt for safeguarding other patients, which also has been described in other studies (Holmberg et al. 2020; Poggenpoel et al. 2011), and by dilemmas around using restrictive measures as restraints or tent beds. These dilemmas reflect experiences of moral distress. Furthermore, participants expressed greater empathy when symptoms were attributed to somatic causes, and less when they were linked to psychiatric disorders. This aligns with the attribution theory, which posits that perceptions of controllability influence compassion and willingness to help (Corrigan et al. 2003). When behaviour is perceived as beyond the patient's control, it tends to elicit greater understanding and support. In contrast, when behaviour is seen as within the patient's control, compassion and willingness to help often decrease. Such attributions illustrate how stigma can shape responses to psychiatric comorbidity, highlighting the need for targeted training in reframing those responses.

The second theme highlights the structural conditions that shape care delivery. Participants described difficulties in balancing time between patients, protecting the safety of all patients and working in environments that were often unsuited for patients with mental health needs. Prior studies have reported similar concerns, including open ward layouts, limited safety adaptations and competing care demands (Holmberg et al. 2020; Poggenpoel et al. 2011). This illustrates that challenges are not simply about taking care of patients' behaviour but also navigating systemic limitations that restrict nursing care. The Dutch context adds an important dimension. Long waiting times for psychiatric care (Netherlands Court of Audit 2020) increasingly result in the admission of patients with psychiatric conditions to general wards. Such systemic issues magnify the strain on general ward staff and underline the urgency of integrating psychiatric expertise and resources into hospital settings.

The third theme demonstrates how nurses sought to develop competence in psychiatric care through knowledge acquisition, experiential learning and multidisciplinary collaboration. The limited mental health training in general nursing education, combined with a strong biomedical focus in hospital culture, left many nurses underprepared for psychiatric aspects of care. These findings are consistent with previous research showing that stigma, low mental health literacy and a lack of confidence contribute to suboptimal care (Alexander et al. 2016; Giandinoto and Edward 2015). The value of psychiatric consultation teams was repeatedly emphasised, indicating the importance of interprofessional collaboration in bridging knowledge gaps. At the same time, collaboration with physicians was described as inconsistent: in some cases, physicians readily engaged in psychiatric consultations, whereas in others participants felt that their concerns about patient behaviour were not fully recognised. To our knowledge, no other qualitative studies have emphasised the value of multidisciplinary collaboration with physicians in general wards as prominently.

Taken together, the findings suggest that caring for patients with medical‐psychiatric comorbidities requires more than individual coping strategies. It demands organisational support and interprofessional approaches. Practically, the findings call for initiatives such as simulation‐based education, which has been shown to improve knowledge, attitudes and confidence in psychiatric care (Fernando et al. 2017). Embedding interprofessional training and support structures could reduce emotional strain, improve collaborative practice and ultimately enhance both nurse well‐being and patient outcomes.

While much research has focused on describing nurses' experiences, the next step should be to evaluate interventions aimed at addressing these challenges.

Future research should examine the implementation and long‐term effects of such interventions. Longitudinal studies could assess its effect on nurses' competence, job satisfaction and patient outcomes. Additionally, research should explore how communication strategies can enhance collaboration between nurses and other professionals, particularly physicians, to support effective teamwork and patient care.

## 6 Strengths and Limitations

This study has several strengths and limitations. Strengths include adherence to COREQ guidelines (Tong et al. 2007), the use of semi‐structured interviews that balanced flexibility and consistency (Braun and Clarke 2013), and the inclusion of a varied sample in terms of sex, age, clinical specialty and years of experience. Sixteen participants provided rich and detailed data, and thematic saturation appeared to have been reached since the last four interviews yielded no substantially new insights.

A potential limitation is the relatively short duration of interviews, which likely reflects nurses' workload during shifts. Additionally, social desirability bias may have occurred, though the interviewer's background as a nurse in a medical‐psychiatric unit may also have facilitated openness and mutual understanding. Finally, while we operationalised saturation as our sampling criterion, we acknowledge that the concept of information power (Malterud et al. 2016) may offer a stronger guiding framework for future qualitative research.

## 7 Conclusion

This study provides new insights into nurses' experiences of caring for patients with medical‐psychiatric comorbidities in general hospital wards. Three themes emerged from the interviews: managing emotions, adapting to contextual factors, and building competence. Nurses reported knowledge gaps and uncertainty, indicating the need for education and stronger interprofessional collaboration. Beyond individual competence, the study underscores the importance of systemic, educational and collaborative support in strengthening care delivery.

## 8 Relevance for Clinical Practice

Caring for patients with medical‐psychiatric comorbidities places significant professional demands on nurses. Supporting nurses requires interventions that reduce emotional burden and enhance competence. Structured opportunities for reflection, targeted education and simulation‐based training can strengthen skills and confidence. In addition, closer collaboration with physicians and psychiatric specialists, together with adjustments in ward organisation, may foster safer, more supportive and person‐centred care.

## Author Contributions

**Ruth Jong:** data collection, data analysis and writing. **Sharon van den Berg:** contribution to discussion and review of the final manuscript. **Sigrid C. J. M. Vervoort:** methodological guidance, thematic framework development, manuscript revision and critical feedback. **Monique van Dijk:** supervision, methodological guidance and feedback throughout all research phases. All authors are in agreement with the manuscript.

## Ethics Statement

Ethical approval for this study was obtained from the Ethics Committee (Approval No. MEC‐2024‐0010).

## Consent

Written informed consent was obtained from all participants.

## Conflicts of Interest

The authors declare no conflicts of interest.

## Data Availability

The data that support the findings of this study are available from the corresponding author upon reasonable request.

## Appendix A

**TABLE A1:** Interview guide.

Topic	Questions
Introduction	– Thank the participant for their time and willingness to participate – Explain the purpose of the study – Mention the estimated duration of the interview (15–30 min) – Guarantee anonymity and confidentiality – Ask for consent to use direct quotes in the research report – Request the participant to sign the informed consent form – Explicitly state that the participant can stop the interview at any time if they wish – Ask for permission to audio‐record the interview – Emphasise that there are no right or wrong answers and that no judgement will be made regarding their responses
Background	– How did you get into nursing? – Can you tell me about your current role? – Do you have any specialisations?
Experiences	– Have you had experiences at work with somatic patients who also had a psychiatric disorder? Could you tell me more about that? – How was this experience for you? How did you behave in this situation? How did you feel? – What types of behaviour do you find difficult to deal with? What interventions do you use in response to such behaviour? – How is the collaboration within your team when dealing with patients who exhibit challenging behaviour? – How is the collaboration with physicians on the ward with patients who exhibit challenging behaviours?
Barriers	– What do you find challenging about caring for patients with a psychiatric disorder?
Facilitators	– What helps you in your work when caring for patients with a psychiatric disorder?
Changes	– What changes would you like to see in your work regarding the care of psychiatric patients? – In what way would you feel better equipped or more confident when working with psychiatric patients?

**TABLE A2:** Coding tree.

Categorising medical‐psychiatric patients	Psychiatric background
	Medical cause
	Disruptive behaviour
Contextual factors	Architectural influences
	Family and relatives
	Dividing time and attention
	Need for multidisciplinary collaboration
	Right place for medical‐psychiatric patients
Attitude health professional	Personality
	Feelings in interaction with the patient
	Focus on medical illnesses
	Putting behaviour in perspective
Knowledge and experience	Influence of knowledge
	Need for more knowledge and tools
	Different interventions
